# P-1264. Explore The Pharmacokinetic/pharmacodynamics Target Attainment (PTA) of cefoperazone/sulbactam

**DOI:** 10.1093/ofid/ofae631.1446

**Published:** 2025-01-29

**Authors:** Yu-Ting Huang, Chien-Chih Wu, Chih-Fen Huang, Chun-Ta Huang

**Affiliations:** National Taiwan University (NTU) Hospital, Taipei, Taipei, Taiwan (Republic of China); National Taiwan University (NTU) Hospital, Taipei, Taipei, Taiwan (Republic of China); National Taiwan University (NTU) Hospital, Taipei, Taipei, Taiwan (Republic of China); National Taiwan University (NTU) Hospital, Taipei, Taipei, Taiwan (Republic of China)

## Abstract

**Background:**

Cefoperazone/sulbactam (CPZ/SUL) are broad-spectrum antibiotics. Current dosage guidelines consider the renal elimination characteristics of SUL. However, with the risk of antimicrobial resistance, whether current dosage recommendation of CPZ/SUL achieves the desired probability of target attainment (PTA) is unclear. The purpose of this study is to compare the PTA between adjusted-dose (AD) CPZ/SUL and standard-dose (SD) CPZ/SUL therapy.

**Figure:**
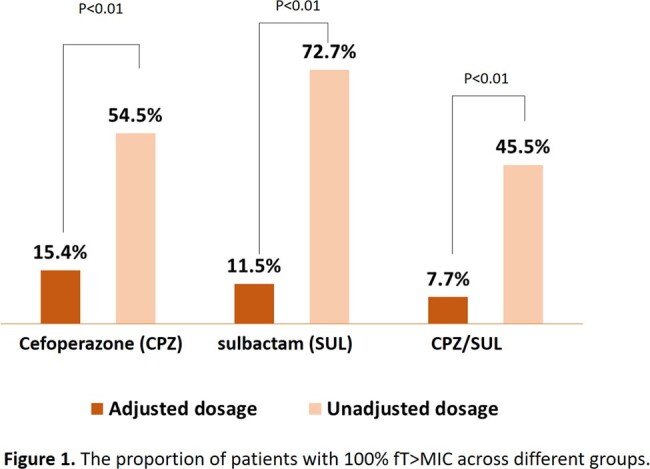

**Methods:**

This observational study was conducted in a tertiary hospital in Taiwan during November 2023 to March 2024. Patients using CPZ/SUL (1:1 ratio) were prospectively enrolled. Two blood samples were collected during the dosing interval to measure CPZ and SUL concentrations by using ultra-high performance liquid-chromatography. Concentrations at the middle of the dosing interval (Cm) and before dosing (Ct) were calculated based on measured concentration. PTA was represented by the fraction of time the free concentration was above the minimum inhibitory concentration (fT >MIC). 50% fT >MIC was defined as Cm >MIC, and 100% fT >MIC was defined as Ct >MIC. AD group was defined as reduced CPZ/SUL doses according to the labeling, while SD group had no dose adjustment despite renal impairments.

**Results:**

A total of 37 patients were enrolled (59.5% male, mean age, 75.4±16.0 years). The most common indication of CPZ/SUL was pneumonia (83.8%). There were 26 patients (70.3%) in the AD group and 11 patients (29.7%) in the SD group. The proportion of patients with 100% fT >MIC was lower in the AD group, as compared to the SD group (CPZ: 15.4% versus 54.5%, P< 0.01; SUL: 11.5% versus 72.7%, P< 0.01). Additionally, 7.7% of patients in the AD group compared to 45.5% of patients in the SD group achieved 100% fT >MIC for both CPZ and SUL (P< 0.01). Thirty patients (81.1%) received weekly vitamin K to prevent CPZ-related coagulopathy, and none had an international normalized ratio above 2.

**Conclusion:**

PTA was inadequate in patients receiving CPZ/SUL based on current dosage adjustment recommendations. Future investigation is needed to determine if inadequate PTA is linked to worse clinical outcomes, especially in resistant pathogens.

**Disclosures:**

**All Authors**: No reported disclosures

